# Supplementary material for: Exploration of influenza incidence prediction model based on meteorological factors in Lanzhou, China, 2014–2017
Source: PLoS One. 2022 Dec 15;17(12):e0277045. doi: 10.1371/journal.pone.0277045 (PMC9754291; doi:10.1371/journal.pone.0277045)
Supplement: S1 Fig — (DOCX) [file pone.0277045.s001.docx]

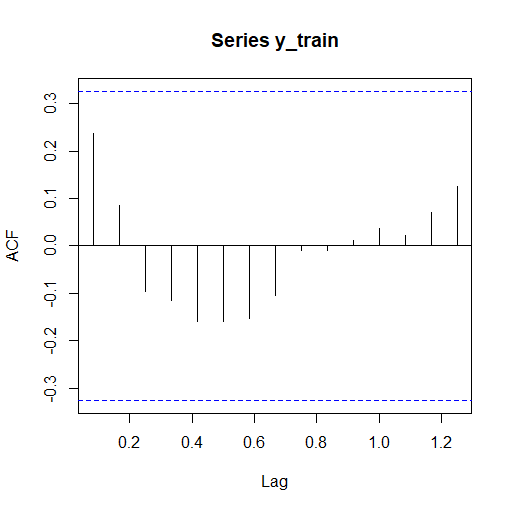

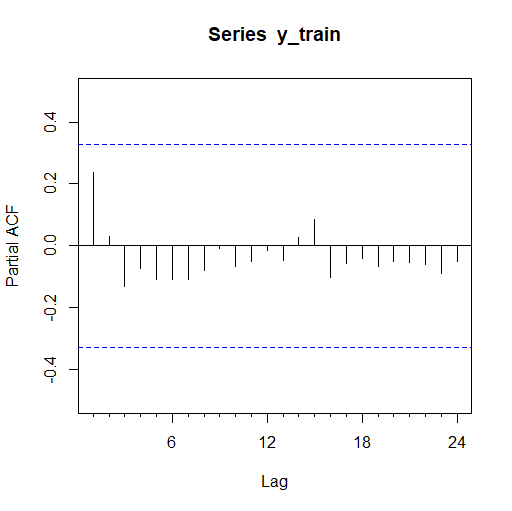


**S1 Fig Autocorrelation (ACF) and Partial Autocorrelation (PACF) graphs of influenza data training sets**
