# Supplementary material for: Exploration of influenza incidence prediction model based on meteorological factors in Lanzhou, China, 2014–2017
Source: PLoS One. 2022 Dec 15;17(12):e0277045. doi: 10.1371/journal.pone.0277045 (PMC9754291; doi:10.1371/journal.pone.0277045)
Supplement: S1 Table — (DOCX) [file pone.0277045.s002.docx]

S1 Table Meteorological factors were incorporated into the ARIMA(1,0,0)(1,0,1)[12]+X multivariable mode

|  | Model | RMSE | MAE | MAPE | AIC | BIC | *P^。^* |
| --- | --- | --- | --- | --- | --- | --- | --- |
| 1 | ARIMA + P | 100.68 | 46.91 | 116.13 | 446.34 | 455.84 | 0.9789 |
| 2 | **ARIMA + T** | **98.05** | **41.90** | **83.69** | **441.34** | **453.84** | **0.9695** |
| 3 | ARIMA + RH | 94.44 | 53.88 | 173.02 | 444.99 | 454.5 | 0.9538 |
| 4 | ARIMA + pre | 93.37 | 51.85 | 152.57 | 445.01 | 454.51 | 0.9797 |
| 5 | ARIMA + Wsd | 102.45 | 52.63 | 142.19 | 447.67 | 457.17 | 0.9516 |
| 6 | ARIMA + Sun | 102.51 | 53.93 | 154.19 | 447.77 | 457.27 | 0.9543 |
| 7 | ARIMA + P + T | 94.32 | 39.98 | 76.71 | 445.79 | 456.87 | 0.9575 |
| 8 | ARIMA + P + RH | 78.00 | 41.98 | 139.11 | 441.44 | 452.53 | 0.9919 |
| 9 | ARIMA + P + pre | 94.28 | 49.63 | 139.19 | 446.83 | 457.92 | 0.9779 |
| 10 | ARIMA + P + Wsd | 96.32 | 46.55 | 131.67 | 447.62 | 458.7 | 0.9920 |
| 11 | ARIMA + P + Sun | 93.53 | 44.88 | 133.93 | 446.96 | 458.04 | 0.9912 |
| 12 | ARIMA + T + RH | 81.19 | 40.76 | 121.82 | 441.69 | 452.78 | 0.9994 |
| 13 | ARIMA + T + Pre | 94.63 | 42.83 | 92.81 | 445.87 | 456.95 | 0.9886 |
| 14 | ARIMA + T + Wsd | 88.59 | 39.78 | 94.61 | 444.81 | 455.89 | 0.9860 |
| 15 | ARIMA + T + Sun | 84.94 | 37.86 | 81.35 | 577.08 | 588.3 | 0.9788 |
| 16 | ARIMA + RH + pre | 85.04 | 49.15 | 166.94 | 444.52 | 455.59 | 0.9958 |
| 17 | ARIMA + RH + Wsd | 83.04 | 50.02 | 156.71 | 445.51 | 455.79 | 0.9858 |
| 18 | ARIMA + RH + Sun | 87.26 | 49.22 | 158.90 | 444.33 | 455.42 | 0.9746 |
| 19 | ARIMA + pre + Wsd | 92.95 | 52.52 | 158.12 | 446.98 | 458.06 | 0.9829 |
| 20 | ARIMA + pre + Sun | 89.71 | 52.31 | 171.47 | 446.46 | 457.54 | 0.9931 |
| 21 | ARIMA + Wsd+ Sun | 84.94 | 37.87 | 81.36 | 577.08 | 588.30 | 0.9788 |
| 22 | ARIMA + P + T + RH | 84.94 | 37.87 | 81.36 | 577.08 | 588.30 | 0.9788 |
| 23 | ARIMA + P + T + pre | 94.75 | 43.04 | 108.32 | 446.17 | 457.28 | 0.9949 |
| 24 | ARIMA + P + T + Wsd | 92.46 | 40.68 | 86.59 | 447.24 | 459.91 | 0.9449 |
| 25 | ARIMA + P + T + Sun | 88.89 | 41.82 | 119.24 | 445.67 | 458.34 | 0.9849 |
| 26 | ARIMA + P + RH + pre | 77.99 | 41.96 | 138.87 | 443.44 | 456.11 | 0.9918 |
| 27 | ARIMA + P + RH + Wsd | 77.81 | 41.63 | 136.47 | 443.40 | 456.07 | 0.9965 |
| 28 | ARIMA + P + RH + Sun | 77.90 | 42.03 | 138.95 | 443.45 | 456.11 | 0.9909 |
| 29 | ARIMA + P + pre + Wsd | 90.48 | 48.71 | 149.62 | 448.16 | 460.83 | 0.9900 |
| 30 | ARIMA + P + pre + Sun | 85.02 | 47.36 | 157.73 | 446.78 | 459.44 | 0.9936 |
| 31 | ARIMA + P + Wsd + Sun | 90.47 | 46.13 | 145.88 | 448.30 | 460.97 | 0.9714 |
| 32 | ARIMA + T + RH + pre | 81.26 | 40.84 | 122.13 | 443.69 | 456.36 | 0.9994 |
| 33 | ARIMA + T + RH + Wsd | 85.26 | 39.14 | 118.25 | 454.97 | 466.12 | 0.9984 |
| 34 | ARIMA + T + RH + Sun | 81.42 | 40.42 | 119.35 | 443.63 | 456.3 | 0.9974 |
| 35 | ARIMA + T + pre + Wsd | 87.68 | 42.62 | 112.13 | 446.47 | 459.14 | 0.9938 |
| 36 | ARIMA + T + pre + Sun | 84.94 | 37.87 | 81.36 | 577.08 | 588.30 | 0.9788 |
| 37 | ARIMA + T + Wsd+ Sun | 84.94 | 37.87 | 81.36 | 577.08 | 588.30 | 0.9788 |
| 38 | ARIMA + RH + pre + Wsd | 81.29 | 37.04 | 124.71 | 444.03 | 491.03 | 0.9198 |
| 39 | ARIMA + RH + pre + Sun | 84.41 | 48.58 | 162.27 | 445.83 | 458.50 | 0.9870 |
| 40 | ARIMA + RH + Wsd + Sun | 84.41 | 48.58 | 162.27 | 445.83 | 458.50 | 0.9870 |
| 41 | ARIMA + pre + Wsd + Sun | 89.01 | 51.12 | 166.74 | 448.28 | 460.95 | 0.9915 |
| 42 | ARIMA + P + T + RH + pre | 94.24 | 42.87 | 112.12 | 455.5 | 465.07 | 0.9711 |
| 43 | ARIMA + P + T + RH + Wsd | 89.46 | 46.32 | 151.45 | 447.75 | 452.32 | 0.9232 |
| 44 | ARIMA + P + T + RH + Sun | 89.01 | 51.12 | 166.74 | 448.28 | 460.95 | 0.9915 |
| 45 | ARIMA + P + RH + pre + Wsd | 77.84 | 41.63 | 136.28 | 445.40 | 459.65 | 0.9963 |
| 46 | ARIMA + P + RH + pre + Sun | 77.94 | 42.07 | 139.16 | 445.44 | 459.69 | 0.9920 |
| 47 | ARIMA + P + pre + Wsd + Sun | 84.37 | 46.46 | 156.32 | 448.54 | 462.79 | 0.9850 |
| 48 | ARIMA + T + RH + pre + Wsd | 84.94 | 37.86 | 81.35 | 577.08 | 588.3 | 0.9788 |
| 49 | ARIMA + T + RH + pre + Sun | 84.37 | 46.46 | 156.32 | 448.54 | 462.79 | 0.9850 |
| 50 | ARIMA + T + pre + Wsd + Sun | 82.95 | 41.60 | 123.38 | 446.52 | 460.77 | 0.9887 |
| 51 | ARIMA + RH + pre + Wsd + Sun | 82.95 | 41.60 | 123.38 | 446.52 | 460.77 | 0.9887 |
| 52 | ARIMA + P + T + RH + pre + Wsd | 78.62 | 40.45 | 129.00 | 447.26 | 463.1 | 0.9943 |
| 53 | ARIMA + P + T + RH + pre + Sun | 82.95 | 41.60 | 123.38 | 446.52 | 460.77 | 0.9887 |
| 54 | ARIMA + P + RH + pre + Wsd + Sun | 82.95 | 41.60 | 123.38 | 446.52 | 460.77 | 0.9887 |
| 55 | ARIMA + T + RH + pre + Wsd + Sun | 82.95 | 41.60 | 123.38 | 446.52 | 460.77 | 0.9887 |
| 56 | ARIMA + P + T + RH + pre + Wsd + Sun | 89.94 | 41.69 | 113.69 | 451.21 | 468.63 | 0.9888 |
