# Supplementary material for: Exploration of influenza incidence prediction model based on meteorological factors in Lanzhou, China, 2014–2017
Source: PLoS One. 2022 Dec 15;17(12):e0277045. doi: 10.1371/journal.pone.0277045 (PMC9754291; doi:10.1371/journal.pone.0277045)
Supplement: Attachment — Submitted filename: Reply to listing.docx [file pone.0277045.s004.docx]

Dear Editors and Reviewers:

Hope you are doing well.

Firstly, thank you for your e-mail and the reviewers’ comments concerning about our manuscript entitled “Exploration of influenza incidence prediction model based on meteorological factors in Lanzhou, China, 2014-2017” (ID: PONE-D-21-37259). Those comments are all valuable and very helpful for revising and improving our paper, as well as the important guiding significance to our researches. Then, we have studied comments carefully and have made correction which we hope to meet with approval. Revised portion are marked in blue in the paper. The main corrections in the paper and the responds to the reviewer’s comments are as follows:

Respond to the Reviewer #1’s comments:

1. 1. L105-107. Who was responsible for case report, only the public health organization? Or both public and private health agencies? How was the influenza case diagnosed (i.e. diagnostic criteria)?

√ Response: 1. **Responsibility reporter:** The data used in this study were downloaded from the disease surveillance network and reported by sentinel hospitals (such as Gansu Provincial People's Hospital, Lanzhou University First Hospital, Lanzhou University Second Hospital, Chengguan District Hospital, etc.).

2. Influenza sentinel surveillance agencies are mainly made up of public agencies, and no private agency is currently tasked with this task.

3. We have clarified the diagnostic criteria for influenza cases in line 116-127 of the article.

**Line 112-122:** The influenza cases in this study are confirmed diagnostic cases, and the diagnostic criteria are:

Clinical manifestations of influenza (mainly fever, headache, myalgia and onset of general discomfort, body temperature up to 39-40 ºC, accompanied by systemic muscle and joint pain, fatigue, loss of appetite and other systemic symptoms, often sore throat, dry cough), positive results of one or more of the following etiological tests:

1. Positive nucleic acid test of influenza virus (real-time RT-PCR and RT-PCR methods can be used).

2. Influenza virus isolation and culture were positive.

3. The level of influenza virus specific IgG antibody in both acute and convalescent sera increased by 4 or more times.

2. L 107, What does the “reviewed” mean?

√ Response: "Reviewed" refers to laboratory etiological diagnosis of influenza-like cases reported by sentinel hospitals by municipal Center for Disease Control and Prevention, and positive cases are confirmed cases of influenza. We have made a corresponding supplement in line 110-112 of the article.

**Line 109-111:** (cases confirmed after laboratory etiological diagnosis of influenza-like cases reported from sentinel hospitals by Lanzhou Center for Disease Control and Prevention).

3. L136-138. It remains unclear that how the meteorological factors were included in the ARIMAX model. Did one model contain only one meteorological variable, or all variables were included in a model at the same time? The collinearity of meteorological factors should be considered if they were combined and included into models.

√ Response: We are very sorry for not showing the results of the 56 ARIMAX models fitted. In the establishment of ARIMAX models, meteorological factors were successively incorporated into the model. Compare the fit and prediction effects of ARIMAX models under different combinations of meteorological factors. We put together these 56 different models into Schedule 1.

Schedule 1 Meteorological factors were incorporated into the ARIMA(1,0,0)(1,0,1)[12]+X multivariable mode

|  | Model | RMSE | MAE | MAPE | AIC | BIC | *P^。^* |
| --- | --- | --- | --- | --- | --- | --- | --- |
| 1 | ARIMA + P | 100.68 | 46.91 | 116.13 | 446.34 | 455.84 | 0.9789 |
| 2 | **ARIMA + T** | **98.05** | **41.90** | **83.69** | **441.34** | **453.84** | **0.9695** |
| 3 | ARIMA + RH | 94.44 | 53.88 | 173.02 | 444.99 | 454.5 | 0.9538 |
| 4 | ARIMA + pre | 93.37 | 51.85 | 152.57 | 445.01 | 454.51 | 0.9797 |
| 5 | ARIMA + Wsd | 102.45 | 52.63 | 142.19 | 447.67 | 457.17 | 0.9516 |
| 6 | ARIMA + Sun | 102.51 | 53.93 | 154.19 | 447.77 | 457.27 | 0.9543 |
| 7 | ARIMA + P + T | 94.32 | 39.98 | 76.71 | 445.79 | 456.87 | 0.9575 |
| 8 | ARIMA + P + RH | 78.00 | 41.98 | 139.11 | 441.44 | 452.53 | 0.9919 |
| 9 | ARIMA + P + pre | 94.28 | 49.63 | 139.19 | 446.83 | 457.92 | 0.9779 |
| 10 | ARIMA + P + Wsd | 96.32 | 46.55 | 131.67 | 447.62 | 458.7 | 0.9920 |
| 11 | ARIMA + P + Sun | 93.53 | 44.88 | 133.93 | 446.96 | 458.04 | 0.9912 |
| 12 | ARIMA + T + RH | 81.19 | 40.76 | 121.82 | 441.69 | 452.78 | 0.9994 |
| 13 | ARIMA + T + Pre | 94.63 | 42.83 | 92.81 | 445.87 | 456.95 | 0.9886 |
| 14 | ARIMA + T + Wsd | 88.59 | 39.78 | 94.61 | 444.81 | 455.89 | 0.9860 |
| 15 | ARIMA + T + Sun | 84.94 | 37.86 | 81.35 | 577.08 | 588.3 | 0.9788 |
| 16 | ARIMA + RH + pre | 85.04 | 49.15 | 166.94 | 444.52 | 455.59 | 0.9958 |
| 17 | ARIMA + RH + Wsd | 83.04 | 50.02 | 156.71 | 445.51 | 455.79 | 0.9858 |
| 18 | ARIMA + RH + Sun | 87.26 | 49.22 | 158.90 | 444.33 | 455.42 | 0.9746 |
| 19 | ARIMA + pre + Wsd | 92.95 | 52.52 | 158.12 | 446.98 | 458.06 | 0.9829 |
| 20 | ARIMA + pre + Sun | 89.71 | 52.31 | 171.47 | 446.46 | 457.54 | 0.9931 |
| 21 | ARIMA + Wsd+ Sun | 84.94 | 37.87 | 81.36 | 577.08 | 588.30 | 0.9788 |
| 22 | ARIMA + P + T + RH | 84.94 | 37.87 | 81.36 | 577.08 | 588.30 | 0.9788 |
| 23 | ARIMA + P + T + pre | 94.75 | 43.04 | 108.32 | 446.17 | 457.28 | 0.9949 |
| 24 | ARIMA + P + T + Wsd | 92.46 | 40.68 | 86.59 | 447.24 | 459.91 | 0.9449 |
| 25 | ARIMA + P + T + Sun | 88.89 | 41.82 | 119.24 | 445.67 | 458.34 | 0.9849 |
| 26 | ARIMA + P + RH + pre | 77.99 | 41.96 | 138.87 | 443.44 | 456.11 | 0.9918 |
| 27 | ARIMA + P + RH + Wsd | 77.81 | 41.63 | 136.47 | 443.40 | 456.07 | 0.9965 |
| 28 | ARIMA + P + RH + Sun | 77.90 | 42.03 | 138.95 | 443.45 | 456.11 | 0.9909 |
| 29 | ARIMA + P + pre + Wsd | 90.48 | 48.71 | 149.62 | 448.16 | 460.83 | 0.9900 |
| 30 | ARIMA + P + pre + Sun | 85.02 | 47.36 | 157.73 | 446.78 | 459.44 | 0.9936 |
| 31 | ARIMA + P + Wsd + Sun | 90.47 | 46.13 | 145.88 | 448.30 | 460.97 | 0.9714 |
| 32 | ARIMA + T + RH + pre | 81.26 | 40.84 | 122.13 | 443.69 | 456.36 | 0.9994 |
| 33 | ARIMA + T + RH + Wsd | 85.26 | 39.14 | 118.25 | 454.97 | 466.12 | 0.9984 |
| 34 | ARIMA + T + RH + Sun | 81.42 | 40.42 | 119.35 | 443.63 | 456.3 | 0.9974 |
| 35 | ARIMA + T + pre + Wsd | 87.68 | 42.62 | 112.13 | 446.47 | 459.14 | 0.9938 |
| 36 | ARIMA + T + pre + Sun | 84.94 | 37.87 | 81.36 | 577.08 | 588.30 | 0.9788 |
| 37 | ARIMA + T + Wsd+ Sun | 84.94 | 37.87 | 81.36 | 577.08 | 588.30 | 0.9788 |
| 38 | ARIMA + RH + pre + Wsd | 81.29 | 37.04 | 124.71 | 444.03 | 491.03 | 0.9198 |
| 39 | ARIMA + RH + pre + Sun | 84.41 | 48.58 | 162.27 | 445.83 | 458.50 | 0.9870 |
| 40 | ARIMA + RH + Wsd + Sun | 84.41 | 48.58 | 162.27 | 445.83 | 458.50 | 0.9870 |
| 41 | ARIMA + pre + Wsd + Sun | 89.01 | 51.12 | 166.74 | 448.28 | 460.95 | 0.9915 |
| 42 | ARIMA + P + T + RH + pre | 94.24 | 42.87 | 112.12 | 455.5 | 465.07 | 0.9711 |
| 43 | ARIMA + P + T + RH + Wsd | 89.46 | 46.32 | 151.45 | 447.75 | 452.32 | 0.9232 |
| 44 | ARIMA + P + T + RH + Sun | 89.01 | 51.12 | 166.74 | 448.28 | 460.95 | 0.9915 |
| 45 | ARIMA + P + RH + pre + Wsd | 77.84 | 41.63 | 136.28 | 445.40 | 459.65 | 0.9963 |
| 46 | ARIMA + P + RH + pre + Sun | 77.94 | 42.07 | 139.16 | 445.44 | 459.69 | 0.9920 |
| 47 | ARIMA + P + pre + Wsd + Sun | 84.37 | 46.46 | 156.32 | 448.54 | 462.79 | 0.9850 |
| 48 | ARIMA + T + RH + pre + Wsd | 84.94 | 37.86 | 81.35 | 577.08 | 588.3 | 0.9788 |
| 49 | ARIMA + T + RH + pre + Sun | 84.37 | 46.46 | 156.32 | 448.54 | 462.79 | 0.9850 |
| 50 | ARIMA + T + pre + Wsd + Sun | 82.95 | 41.60 | 123.38 | 446.52 | 460.77 | 0.9887 |
| 51 | ARIMA + RH + pre + Wsd + Sun | 82.95 | 41.60 | 123.38 | 446.52 | 460.77 | 0.9887 |
| 52 | ARIMA + P + T + RH + pre + Wsd | 78.62 | 40.45 | 129.00 | 447.26 | 463.1 | 0.9943 |
| 53 | ARIMA + P + T + RH + pre + Sun | 82.95 | 41.60 | 123.38 | 446.52 | 460.77 | 0.9887 |
| 54 | ARIMA + P + RH + pre + Wsd + Sun | 82.95 | 41.60 | 123.38 | 446.52 | 460.77 | 0.9887 |
| 55 | ARIMA + T + RH + pre + Wsd + Sun | 82.95 | 41.60 | 123.38 | 446.52 | 460.77 | 0.9887 |
| 56 | ARIMA + P + T + RH + pre + Wsd + Sun | 89.94 | 41.69 | 113.69 | 451.21 | 468.63 | 0.9888 |

4. L140-143 “the first 36 months (2014/01-2016/12) were used to construct the time series model”. If the monthly data was used to construct the model, it should be clearly indicated that how the daily data was aggregated into monthly data.

√ Response: We are very sorry for not being clear about this area. The processing of data was supplemented on lines 129-141.

**Line 128-140:**

**Data preprocessing**

The "date of case onset" in the influenza case data was used as a single variable frequency count to obtain the number of cases on a daily basis from January 1, 2014 to December 31, 2017. If there was no case onset on a certain day in a certain year or month, the observed number of cases on that day was recorded as 0. For the missing meteorological data, the mean value of adjacent points was used to repair the data. Finally, the daily incidence data and daily meteorological data were matched one by one. Excel 2010 software was used to integrate the data, establish a database and form a time series file.

Because of the low number of influenza cases reported daily, the influenza cases and meteorological factors data should be converted to monthly data during the establishment of SARIMA(X) model: the influenza cases are the sum of influenza cases per month, and meteorological factors are the monthly average of daily data.

5. L199. For the ADF test, P>0.001 could not indicate that the original sequence was stationary. The exact P-value should be given, and it should be compared with 0.05 to prove the stationary.

√ Response: We are very sorry for the mistake in our sentence and “*P >* 0.001” have been changed to " *P* < 0. 001" in line 233 of the text.

6. Response to comment: Fig 2 and Table 5 just provided the same information.

√ Response: Yes, Figure 2 and Table 5 showed the same information, but they have different effects. Figure 2 is to compare the fitting effects of the two models more intuitively, and Table 5 is to give the exact fitting values of the two models.

7. Please compare both the fitting and forecasting performance (e.g. RMSE) of these two models.

√ Response: The fitting and forecasting effects of the two models have been compared in original text. The univariate models were shown in Table 4, and the multivariate model was shown in lines 250-251.

Univariate models:

**Table 4 Selection of the univariate ARIMA model**

| Model | RMSE | MAE | MAPE | AIC | BIC | *P^。^* |
| --- | --- | --- | --- | --- | --- | --- |
| (1,0,0)(1,0,1)[12] | 102.58 | 53.82 | 151.36 | 445.80 | 453.72 | 0.9588 |
| (0,0,1)(1,0,1) [12] | 103.00 | 53.62 | 152.30 | 446.08 | 454.00 | 0.8939 |
| (1,0,1)(1,0,1)[12] | 102.57 | 53.92 | 152.02 | 447.79 | 457.29 | 0.9760 |
| (1,0,0)(1,0,2)[12] | 102.58 | 53.83 | 151.46 | 447.8 | 457.3 | 0.9586 |
| (0,0,1)(1,0,2) [12] | 103.00 | 53.61 | 152.24 | 448.08 | 457.58 | 0.8943 |
| (1,0,1)(1,0,2)[12] | 102.94 | 55.06 | 166.87 | 450.85 | 461.94 | 0.1157 |

Multivariate model:

**Lines 250-251:** ARIMA (1,0,0)(1,0,1)[12] with daily average temperature (T-mean) as external predictors was the optimal ARIMAX model, with a minimum AIC = 441.34 and a minimum BIC = 453.84, (RMSE = 98.05, MAE = 41.90, MAPE = 83.69, *P* = 0.9695).

8. There was an influenza outbreak in 2016, while the other years showed slight fluctuation. This outbreak had a certain impact on the modelling performance. Thus, if there is no such outbreak, that is, the number of influenza cases changes slightly from year to year, does the ARIMAX model still have high performance? Since influenza outbreak is not common in Lanzhou, the abovementioned scenario should be considered.

√ Response: We took the average number of influenza cases in 2014 and 2015 as influenza cases from January to December in 2016 to reduce the fluctuation of data and fit the SARIMA(1,0,0)(1,0,1)[12] + T-mean model. The fitting results are shown in the figure below. The results showed that the fitting effect was worse, indicating that the fluctuation in 2016 had little impact on the prediction model, because the reported cases of influenza showed an upward trend from 2014 to 2017, and it was normal to have fluctuations from year to year.

9. How much data could the ARIMA/ARIMAX model predict? From Fig 2 and Table 5, the models showed relatively good predicting performance during the first half of 2017, but significant forecast error could be found in the second half of 2017. What might be the reasons?

√ Response: In this study, we used 3 years of data for the fitting model, which can predict 12 months, and its prediction from January to October was better. The reason for the poor effect of late prediction is that the number of influenza cases in winter of 2017 is higher than that of previous years, which leads to the low prediction result of the model.

10. Please discuss whether this ARIMAX model is applicable in other conditions (e.g. in other regions).

√ Response: We have discussed in line 323-327 whether the ARIMAX model is applicable in other regions.

For other parts of Gansu Province that are similar to Lanzhou's geographical environment, meteorological factors, economic conditions and other factors, we can also try to use this model to predict local influenza cases. For areas with large differences from Lanzhou (such as Beijing, Shanghai, etc.), we should explore appropriate ARIMAX models based on the influencing factors of influenza activity in other regions to predict local influenza activity.

11. Please revise the language of this manuscript. And some typos need to be corrected.

√ Response: We feel very sorry for some spelling mistakes. We have checked and revised the full text and marked it in blue font.

Respond to the Reviewer #3’s comments:

1. The description of daily incidence data could be clearer, as the “reviewed cases” has an ambiguous meaning.

√ Response: "Reviewed cases " refers to laboratory etiological diagnosis of influenza-like cases reported by sentinel hospitals by municipal Center for Disease Control and Prevention, and positive cases are confirmed cases of influenza. We have made a corresponding supplement in line 110-112 of the article.

**Line 109-111:** (cases confirmed after laboratory etiological diagnosis of influenza-like cases reported from sentinel hospitals by Lanzhou Center for Disease Control and Prevention).

2. Why the parameter of the seasonal period is set to be 12?

√ Response: Because we used monthly data for forecast fitting, the period is 12 months.

3. Maybe “Seasonal ARIMA” is a more accurate word to describe the model instead of ARIMA.

√ Response: Thank you very much for your suggestion. In this paper, we actually used the seasonal ARIMA model. We have changed ARIMA(X) to SARIMA(X) as shown in the blue font.

4. It would be better to clarify the formula of the seasonal ARIMA model in the method part.

√ Response: We feel very sorry for not giving the formula of ARIMA model, which we added in line 152-157 of the article.

**Line 152-157:** The equation of the model is as follows:

$$\phi\left( B \right)\varphi\left( B^{s} \right)\left( 1-B \right)^{D}Y_{t}=\theta\left( B \right)\Theta\left( B^{s} \right)\Sigma_{t}$$

Where$Y_{t}$is the predicted value of the time series model at time t, B is the backshift operator, $\phi\left( B \right)$ is the autoregressive operator, $\varphi\left( B^{s} \right)$ is the seasonal autoregressive operator, and $\theta\left( B \right)$ is the shift Average operator, $\Theta\left( B^{s} \right)$ is the seasonal moving averag e operator, and $\Sigma_{t}$ is the random error.

5. Both the ACF and PACF plots do not have a significant spike, could you discuss this limitation, and does it matter to your model?

√ Response: ACF and PACF plots are used to determine model parameters and whether they are seasonal. Both ACF and PACF values within two standard deviations have little effect on our model. By reviewing the literature, we found that the ACF and PACF plots of the original sequences of certain variables in which Irie^[1]^, Ilie^[2]^ and Dou^[3]^ were all within two standard deviations when establishing ARIMA model.

[1] Ilie OD, Cojocariu RO, Ciobica A, Timofte SI, Mavroudis I, Doroftei B. 2020. Forecasting the Spreading of COVID-19 across Nine Countries from Europe, Asia, and the American Continents Using the ARIMA Models. Microorganisms. 30;8(8):1158. doi: 10.3390/microorganisms8081158. PMID: 32751609; PMCID: PMC7463904.

[2] Ilie OD, Ciobica A, Doroftei B. 2020. Testing the Accuracy of the ARIMA Models in Forecasting the Spreading of COVID-19 and the Associated Mortality Rate. Medicina (Kaunas). 56(11):566. doi: 10.3390/medicina56110566. PMID: 33121072; PMCID: PMC7694177.

[3] Dou ZW, Ji MX, Wang M, Shao YN. 2022. Price Prediction of Pu'er tea based on ARIMA and BP Models. Neural Comput Appl. 34(5):3495-3511. doi: 10.1007/s00521-021-05827-9. Epub 2021 Mar 16. PMID: 33746365; PMCID: PMC7960402.

6. The AIC value did improve from ARIMA (445.80) and ARIMA(X) (441.34), but didn’t improve much, AIC and BIC indeed are the good methods to evaluate the model fit, but their values are larger when more variables were added. The optimal ARIMA(X) model is selected by the minimum AIC and BIC value, the procedure of comparing the models’ prediction error should also be displayed, which will make the optimal model more convincing.

√ Response: Thanks for your comment. In the modeling process of this paper, we chose the optimal model according to the minimum principle of AIC and BIC. The results show that the fitting effect of the multivariate ARIMAX model established by adding the average temperature as an exogenous variable is better than that of the univariate ARIMA model, which also makes the model more convincing.

Other changes:

1. Line 3-4, We added an author, Sheng Li, who has made outstanding contributions to this paper, and changed him to the second corresponding author. Author Yongjun Li was replaced as the fifth author.
2. Line 10, The second corresponding author's [organization](javascript:;) was added, “^3^ Lanzhou First People's Hospital, Gansu Lanzhou 730000, China”.
3. Line 16, Added the address of the second corresponding author, “^#b^ Lanzhou First People's Hospital, Gansu Lanzhou, China”
4. Line 19, The second corresponding author's [e-mail](javascript:;) was added, “1178708407@qq.com”.
5. Line 43-44, Modified statement “which has strong ability to mutate and fast propagation speed. The population is generally susceptible to influenza virus, causing the epidemic or outbreak”.
6. Line 46, the statement of “influenza outbreaks cause” was corrected as “the outbreaks of influenza can cause”.
7. Line 51-55, “Therefore, many researchers have explored and analyzed the factors that influenced the spread and infection of influenza，including individual genetic differences, changing population demographics, antibiotic resistance and environmental, etc.^[11-14]^.” has been changed to “For the outbreak of influenza, there are many factors (such as individual genetic differences, changing population demographics, antibiotic resistance and environmental, etc.) ^[11-14]^ can affect the spread and infection of flu, therefore, many researchers have explored and analyzed these factors to prevent and control the influenza in some degree.”
8. Line 63, “At present, however” has been changed to “However, at present”.
9. Line 74-76, the statements of “Influenza in Lanzhou occurs seasonally, but there is no study on the prediction of the number of cases per month” were corrected as “Influenza in Lanzhou occurs seasonally, up to now, there are few studies on forecasting the cases of influenza per month.”.
10. Line 77-79, “Thus, the prediction of the cases of influenza in Lanzhou can provide a possibility for preventing and controlling influenza transmission for the government.” was added.
11. Line 93-94, “this study analyzed the characteristics of influenza incidence, and study the impact of meteorological factors on influenza activities in Lanzhou” has been changed to “the characteristics of influenza incidence was analyzed and the impact of meteorological factors on influenza activities in Lanzhou was studied”.
12. Line 96-97, “providing a theory reference for early prevention and control of influenza.” was added.
13. Line 147-149, “Because of the seasonal nature of influenza activity, the seasonal autoregressive integrated moving average (SARIMA) model was used in this study.” was added.
14. Line 211-213, the statement of “Table 3 provides the matrix of Pearson’s correlation coefficients within the meteorological factors” has been changed to “The matrix of Spearman correlation coefficients within the meteorological factors was showed in Table 3”.
15. Line 216-218, the statement of “The correlation coefficient was -0.489 between the influenza cases and temperature, and it was -0.088 between the influenza cases and relative humidity” was corrected as “The correlation coefficient between the number of influenza cases and temperature was -0.489, that between the number of influenza cases and relative humidity was -0.088.”.
16. Line 220, “r” has been changed to “*r*”.
17. Line 245, “daily average atmospheric pressure (P), precipitation (pre), average relative humidity (RH), hours of Sunshine (Sun), daily average temperature (T-mean) and daily average wind speed (wsd)” has been changed to “P, pre, RH, Sun, T-mean and wsd”.
18. Line 255, “was” has been changed to “were”.
19. Line 270-273, “This study mainly analyzed the number of influenza cases in Lanzhou from 2014 to 2017, explored the optimal prediction model, and evaluated whether adding meteorological factors as exogenous variables would have a better prediction effect” has been changed to “In this paper, the influenza cases in Lanzhou from 2014 to 2017 were analyzed, the optimal prediction model was established, and whether adding meteorological factors as exogenous variables would have a better prediction effect was evaluated”.
20. Line 294-295, the statements of “There were also winter peak and spring secondary peak in the epidemic of influenza in Lanzhou. Studies have found that winter influenza epidemics caused considerable morbidity and mortality in temperate regions” were corrected as “This characteristic is consistent with those studies in which that winter influenza epidemics caused considerable morbidity and mortality in temperate regions”.
21. Line 303-304, the statement of “there was a positive correlation between influenza activity and temperature and relative humidity” was corrected as “temperature and relative humidity would impose an increase for the transmission of influenza virus”.
22. Line 316, “the virus diffuses” has been changed to “the diffused virus”.
23. Line 324-328, the statements of “Using ARIMA model, the relationship between the number of influenza cases and meteorological variables makes the prediction model have significant predictive power. This is similar to what the researchers have found elsewhere, which is that adding weather as an exogenous variable was a better predictor of influenza activity” were corrected as “The established SARIMA model in this paper will benefit to predict the influenza cases in Lanzhou based, the correlations between the influenza cases and meteorological variables. This is similar to what the researchers have found elsewhere, when is that adding weather as an exogenous variable, the model was a better predictor of influenza activity”.
24. Line 329-338, We put “Due to limitations in available and reliable data of influenza time-series in Lanzhou, the influenza forecasting accuracy is impaired. Therefore, we should continue to strengthen the epidemiological surveillance of influenza, as far as possible to reduce the failure rate. In addition, there may be differences in viral virulence that have not been considered” the limitations of [article](javascript:;).

Special thanks to you for your good comments.

We tried our best to improve the manuscript and made some changes in the manuscript. These changes will not influence the content and framework of the paper. And here we did not list the changes but marked in blue in revised paper.

We appreciate for Editors and Reviewers’ warm work earnestly, and hope that the correction will meet with approval.

Once again, thank you very much for your comments and suggestions.

Best regards,

Dr.Xiaochun Yin

School of Public Health

Gansu University of Chinese Medicine
