# Supplementary material for: Exploration of influenza incidence prediction model based on meteorological factors in Lanzhou, China, 2014–2017
Source: PLoS One. 2022 Dec 15;17(12):e0277045. doi: 10.1371/journal.pone.0277045 (PMC9754291; doi:10.1371/journal.pone.0277045)
Supplement: Attachment — Submitted filename: Response to Reviewers.docx [file pone.0277045.s005.docx]

Dear Editors and Reviewers:

Hope you are doing well.

Firstly, thank you for your e-mail and the reviewers’ comments concerning about our manuscript entitled “Exploration of influenza incidence prediction model based on meteorological factors in Lanzhou, China, 2014-2017” (ID: PONE-D-21-37259). Those comments are all valuable and very helpful for revising and improving our paper, as well as the important guiding significance to our researches. Then, we have studied comments carefully and have made correction which we hope to meet with approval. Revised portion are marked in blue in the paper. The main corrections in the paper and the responds to the reviewer’s comments are as follows:

Respond to the Reviewer #1’s comments:

1. Please show both the fitting performance and the forecasting performance of both the optimal univariate model and the optimal multi-variate model. The fitting RMSE was calculated by compare the model fitted 2014-2016 data with the actual data in the same time period, while the forecasting RMSE was calculated by compare the predicted and actual data in 2017. Maybe the RMSEs in the Table 4 and Line 205 were the fitting performance? Table 5 showed the results of forecasted 2017 disease incidence, and the RMSE of each month should be shown in this table to indicate the forecasting performance. Also, in Table 5, the model output was the “Predicted” values instead of the “Fitted” values.

√ Response: Yes, the RMSEs, MAEs and MAPEs in Table 4 and line 205 were the fitting performance and the forecasting performance of the univariate models and the optimal multi-variate.

RMSE is used to measure the discreteness of a group of numbers themselves. Suppose there were n training data $x_{i}$, the true output of each training data $x_{i}$was $y_{i}$, and the predicted value of $x_{i}$ by the model was $\hat{y}i$. The RMSE of the model under n training data can be defined as follows ^[1]^:

Therefore, RMSE is the evaluation index of model effect calculated according to each fixed ARIMA(X) model, and not every predicted value will have a corresponding RMSE value. In order to reflect the forecast effect, we also calculate the relative error of each month in Table 5.

In Table 5 and Figure 2, we have modified the “Fitted” values to the “Predicted” values.

[1] Guo Y, Feng Y, Qu F, Zhang L, Yan B, Lv J. Prediction of hepatitis E using machine learning models. PLoS One. 2020 Sep 17;15(9): e0237750. doi: 10.1371/journal.pone.0237750.

**Table 5 Prediction results of SARIMA(X) model on the number of influenza cases in Lanzhou from January 2017 to December 2017**

| Time | Actual | SARIMA(1,0,0)(1,0,1)[12] | | | | | |  | SARIMA(1,0,0)(1,0,1)[12] + T-mean | | | | | | | |  |
| --- | --- | --- | --- | --- | --- | --- | --- | --- | --- | --- | --- | --- | --- | --- | --- | --- | --- |
|  |  | Predicted | Lo 80 | Hi 80 | Lo 95 | Hi 95 | RE(%) | | |  | Predicted | Lo 80 | Hi 80 | Lo 95 | Hi 95 | RE(%) | |
| [Jan](javascript:;). 2017 | 105 | 82.04 | -57.41 | 221.48 | -131.22 | 295.30 | -21.87 | | |  | 112.81 | -22.61 | 248.23 | -94.30 | 319.91 | 7.44 | |
| Feb. 2017 | 35 | 79.43 | -64.10 | 222.87 | -139.94 | 298.81 | 126.94 | | |  | 97.43 | -39.24 | 234.11 | -111.60 | 306.46 | 178.37 | |
| Mar. 2017 | 63 | 130.08 | -13.60 | 273.75 | -89.65 | 349.80 | 106.48 | | |  | 79.63 | -57.07 | 216.33 | -129.44 | 288.70 | 26.40 | |
| Apr. 2017 | 39 | 60.10 | -83.59 | 203.78 | -159.65 | 279.84 | 54.10 | | |  | 53.44 | -83.27 | 190.14 | -155.63 | 262.50 | 37.03 | |
| May. 2017 | 27 | 57.47 | -86.21 | 201.16 | -162.27 | 277.22 | 112.85 | | |  | 33.46 | -103.24 | 170.16 | -175.61 | 242.52 | 23.93 | |
| Jun. 2017 | 15 | 57.02 | -86.66 | 200.71 | -162.72 | 276.77 | 280.13 | | |  | 19.97 | -116.74 | 156.67 | -189.10 | 229.03 | 33.13 | |
| Jul. 2017 | 6 | 57.00 | -86.68 | 200.69 | -162.74 | 276.75 | 850.00 | | |  | 0.61 | -136.09 | 137.31 | -208.46 | 209.68 | -89.83 | |
| Aug. 2017 | 8 | 56.57 | -87.11 | 200.26 | -163.17 | 276.32 | 607.13 | | |  | 20.94 | -115.76 | 157.64 | -188.12 | 230.01 | 161.75 | |
| Sep. 2017 | 17 | 57.60 | -86.09 | 201.28 | -162.15 | 277.34 | 238.82 | | |  | 32.28 | -104.43 | 168.98 | -176.79 | 241.34 | 89.88 | |
| Oct. 2017 | 55 | 58.99 | -84.69 | 202.68 | -160.76 | 278.74 | 7.25 | | |  | 63.50 | -73.20 | 200.20 | -145.57 | 272.57 | 15.45 | |
| Nov. 2017 | 156 | 60.78 | -82.90 | 204.46 | -158.96 | 280.53 | -61.04 | | |  | 90.81 | -45.89 | 227.51 | -118.26 | 299.88 | -41.79 | |
| Dec. 2017 | 236 | 67.50 | -76.18 | 211.19 | -152.24 | 287.25 | -71.40 | | |  | 116.64 | -20.06 | 253.34 | -92.43 | 325.71 | -50.58 | |

2. Line 325-327. Results of this study were compared with other relevant research, and it would be better to show more about the consistency and inconsistency compared with the similar research that conducted in the same/different regions, and the possible reasons for the difference.

√ Response: In order to explain more about the consistency and inconsistency with other studies and the reasons for the differences, we have made an explanation in line 331-342 of the article.

Chadsuthi et al. ^[69]^ studied the influenza prediction model in central and southern Thailand, and found that the ARIMAX model including the average temperature lagging 4 months and the minimum relative humidity lagging 2 months was the model suitable for the central region, while the ARIMAX model including the minimum relative humidity lagging 4 months was the best model suitable for the southern region. In Abidjan, the inclusion of rainfall can improve the performance of influenza fitting and forecasting models ^[70]^. Although most studies have found that the inclusion of meteorological factors can improve the performance of influenza prediction models, due to the differences in regional climatic conditions and human factors, the meteorological factors included in the optimal multivariate models for influenza prediction in each region are different. This is a further reminder of the regional nature of influenza prediction models.

3. Why the plots in Fig 2 had two titles? Please remove one if possible.

√ Response: We are very sorry for the misunderstanding caused by the question in Figure 2, but the two headings in Figure 2 are different contents. In order to avoid misunderstanding, we have modified Figure 2, as shown in Figure 2.

A

B

Fig 2 Prediction fitting of SARIMA(X) model on the number of influenza cases in Lanzhou from January 2017 to December 2017 (A represents the Univariate SARIMA model, B represents the Multivariate SARIMAX model)

4. Some typos: e.g. Line176: 2016/012; Line 210: “Spearman line correlation”; Line 221: “。”; Table 4 please show the values with same number of decimals.

√ Response: We are very sorry for the spelling mistakes in the article. We have been revising the position of the article.

Line 176: the first 36 months (2014/01-2016/12) were used to construct the time series model

Line 210: Spearman correlation analysis was used to analyze the daily influenza cases

Line 221: (*r* = -0.719, *P* < 0.01).

We have unified the number of decimal places in Tables 5. However, in order to keep the consistency of *P* decimal places in Table 4, we still keep four decimal places of *P^。^* in Table 4. After R software were run, AICs and BICs values only display two decimal places, so they cannot keep the same number of decimal places as *P^。^*.

Other changes:

1. Line 3, Since the author of the revision of the article, Hai Zhu, has made a great contribution to the revision of this article, we have decided to work with Hai Zhu as a common author.
2. Line 7, We added a second affiliation of Meixia Du, “Gansu Provincial Cancer Hospital, Gansu Lanzhou 730050, China”.
3. Line 21, We added the notation for the contributed equally to this work, “^¶^ These authors contributed equally to this work”.
4. Line 367, We changed the order of authors' contributions, “Hai Zhu: Methodology, [Data Collection](http://www.baidu.com/link?url=jJb4Q_HKMBkPyhLSfzNg_4AVvtpVicmRut5HWUyDgk-CkBCSpsldExB2BZpEXm5h-yly62j9WO04rk5VmNCv7hTJU378PuATTg30DxsiC6ddu-VIBapvf7V6eM7uPWDg&wd=&eqid=99e8f433002590760000000360608110), Article modification”.

Special thanks to you for your good comments.

We tried our best to improve the manuscript and made some changes in the manuscript. These changes will not influence the content and framework of the paper. And here we did not list the changes but marked in blue in revised paper.

We appreciate for Editors and Reviewers’ warm work earnestly, and hope that the correction will meet with approval.

Once again, thank you very much for your comments and suggestions.

Best regards,

Dr.Xiaochun Yin

School of Public Health

Gansu University of Chinese Medicine
